# Abnormal M1/M2 macrophage phenotype profiles in the small airway wall and lumen in smokers and chronic obstructive pulmonary disease (COPD)

**DOI:** 10.1038/s41598-017-13888-x

**Published:** 2017-10-17

**Authors:** Mathew Suji Eapen, Philip M. Hansbro, Kielan McAlinden, Richard Y. Kim, Chris Ward, Tillie-Louise Hackett, Eugene H. Walters, Sukhwinder Singh Sohal

**Affiliations:** 1 NHMRC Centre for Research Excellence for Chronic Respiratory Disease, School of Medicine, University of Tasmania, Hobart, Tasmania Australia; 2 School of Health Sciences, Faculty of Health, University of Tasmania, Launceston, TAS Australia; 3 Priority Research Centre for Healthy Lungs, Hunter Medical Research Institute and The University of Newcastle, Newcastle, New South Wales Australia; 4 Woolcock Institute of Medical Research, University Technology Sydney, Sydney, New South Wales Australia; 5 Institute of Cellular Medicine, University of Newcastle, Newcastle Upon Tyne, UK; 6 Department of Anesthesiology, Pharmacology & Therapeutics, University of British Columbia, Vancouver, British Columbia, Canada, and UBC Centre for Heart Lung Innovation, St. Paul’s Hospital, Vancouver, British Columbia Canada

## Abstract

We explore potential dysregulation of macrophage phenotypes in COPD pathogenesis through integrated study of human small airway tissue, bronchoalveolar lavage (BAL) and an experimental murine model of COPD. We evaluated human airway tissue and BAL from healthy controls, normal lung function smokers (NLFS), and COPD subjects. Both small airways and BAL cells were immunohistochemically stained with anti-CD68 for total macrophages and with anti-CD163 for M2, and anti-iNOS for M1 macrophages. Multiplex ELISA measured BAL cytokines. Comparable cigarette smoke-induced experimental COPD mouse model was assessed for relevant mRNA profiles. We found an increase in pro-inflammatory M1s in the small airways of NLFS and COPD compared to controls with a reciprocal decrease in M2 macrophages, which remained unchanged among pathological groups. However, luminal macrophages showed a dominant M2 phenotype in both NLFS and COPD subjects. BAL cytokine skewed towards an M2 profile with increase in CCL22, IL-4, IL-13, and IL-10 in both NLFS and COPDs. The mouse-model of COPD showed similar increase in mRNA for M2 markers. Our finding suggests abnormal macrophage switching in both mucosal and luminal areas of COPD patients, that strongly associated with cytokine balance. There may be potential for beneficial therapeutic cytokine manipulation of macrophage phenotypes in COPD.

## Introduction

Airflow limitation is the defining feature of COPD and is due primarily to small airway wall fibrosis, thickening, and luminal narrowing and progressive obliteration. These events occur early in disease even before symptoms appear or lung function changes^1^. Further, emphysema may occur in some COPD individuals, which also adds to airflow limitation^2^. Airway inflammation has become accepted as the key underlying driver of COPD pathophysiological manifestations, but with limited core evidence, at least for the airway wall rather than the lumen^3^. We and others have suggested that the role of inflammation in COPD requires reassessment, especially in earlier stages of disease where rather paradoxically hypo-cellularity^4–6^ and an overall decrease in inflammatory cell numbers^7^ has recently been described in both the large and small airway wall in COPD patients.

Increases in the total numbers of macrophages in the airway lumen have been well established in smokers and COPD patients^8–10^. We recently assessed small airway wall tissue for CD68 + macrophages, and although, we found a greater number of macrophages in the small airway wall in normal controls compared to the large airway, there were no changes in their numbers in smokers or COPD^3,5^.

Macrophages can exhibit polarized phenotypes, with, M1 and M2 subpopulations reflecting the paradigm of Th1 and Th2 lymphocytes^11^. M1 macrophages have been described as cytotoxic and pro-inflammatory, and are characterized by secretion of the cytokines interferon (IFN)-γ and IL-12, and by promoting Th1-type immunity^11–13^. In contrast, M2 macrophages are considered anti-inflammatory and are linked to tissue repair and fibrosis, secreting pro-Th2 cytokines including CCL22, IL-4, IL-13 and IL-10 ^14^.

Macrophages have a fundamental ability to metabolize L-arginine either to nitric oxide (NO) or ornithine, using the mutually substrate-competitive enzymes inducible nitric-oxide synthase (iNOS) or arginase-I (Arg-1), respectively^12^. Phenotypically M1 macrophages exhibit increased iNOS expression, while M2 macrophages are typified by an increase in Arg-1, which promotes collagen synthesis by making the amino acid proline available to fibroblasts^15,16^. The competition between iNOS and Arg-1 for L-arginine can drive contrasting pathologies functionally through opposed macrophage phenotypes^17^.

In the current study, we have characterized the phenotypic and metabolic regulatory dichotomy of airway wall macrophage populations and their micro-environments in human lung tissue and bronchoalveolar lavage (BAL), and related phenotype is switching to smoking, COPD, and lung function. We further confirmed these findings in a six-week cigarette smoke-induced mouse model of experimental COPD.

## Results

### M1/M2 phenotypes in small airways

**Figure 1:**
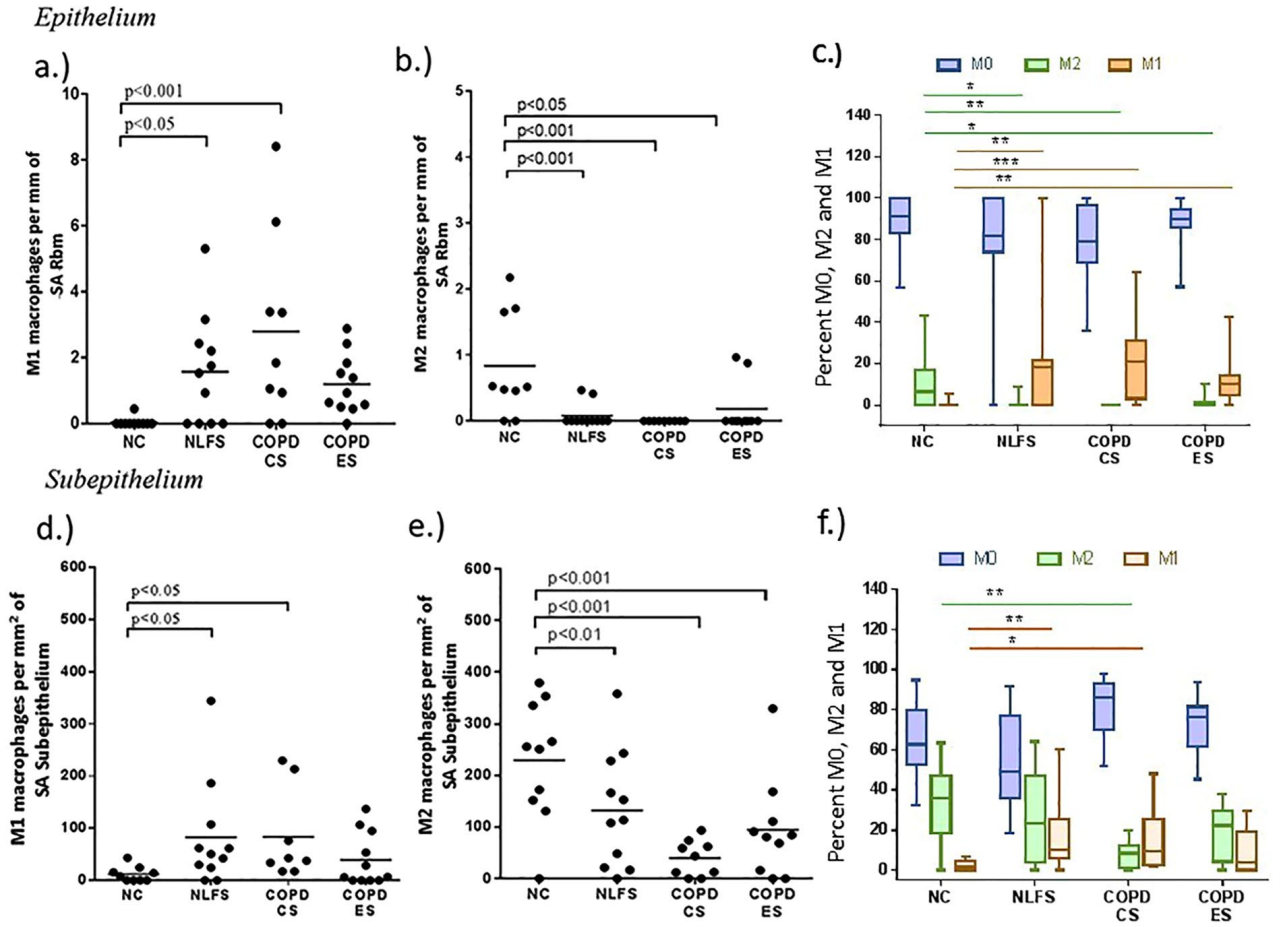
Macrophages phenotype numbers in SA tissue. In epithelium: an increase in the numbers of (**a**) M1 and a decrease in (**b**) M2 macrophages observed in smokers and COPDs compared to NC. Similar pattern was also observed in the sub-epithelium (**d**) M1 and (**e**) M2. Percent change in macrophage phenotypes M0, M1 and M2 in (**c**) Epithelium and (**f**) Sub-epithelium expression (Data in median and range; *p < 0.05, **p < 0.01).

In the small airway epithelium, the dominant macrophage type both in numbers and percentages was the non-differentiated M0, especially in normal control (NC) subjects (Fig. 1a,c), where there were essentially very few M1 (median percenatge 0%; range 0–5.2) macrophages. There was a significant increase in percent M1 population in normal lung function smokers (NLFS) [median percenatge18%; range 0–100; (p < 0.05)] and COPD current smoker (COPD-CS) [median percentage 21.2% range 0.0–64.1; (p < 0.01)], which reverted partially towards normal in COPD ex-smokers (COPD-ES) [median percentage 10% range 0.0–42; (p < 0.05)] (Fig. 1c). Small numbers and percentage of M2 macrophages were present in the NC (median percentage 6.4%; range 0.0–43.3) but were almost absent in NLFS [median percenatge 0%; range 0.0–9.0 (p < 0.05)], COPD-CS [median percentage 0%; range 0.0–0.01 (p < 0.01)] and COPD-ES [median percentage 0%; range 0.0–10.1 (p < 0.05)] (Fig. 1b and c).

In the subepithelium in NC, the M0 population was less dominant than in the epithelium, with relatively fewer M1s (median percentage 1.6%; range 0.0–6.3) and more M2 macrophages (median 36%; range 0.0–63) (Fig. 1f). A significant rise in the M1 population was observed in the NLFS [median percenatge 10.1%; range 0.0–60.2 (p < 0.01)] and COPD-CS [median percenatge 9.6% range 1.6–48 (p < 0.05)]. There were declines compared to normal in the M2 population in smokers (median percentage 23% range 0.0–64.1) and COPD, again especially significant in COPD-CS [median percentage 8.4%;range 0.0–19.9 (p < 0.01)] (Fig. 1f). No statistical significant difference was observed between NLFS and COPD-CS.

**Figure 2:**
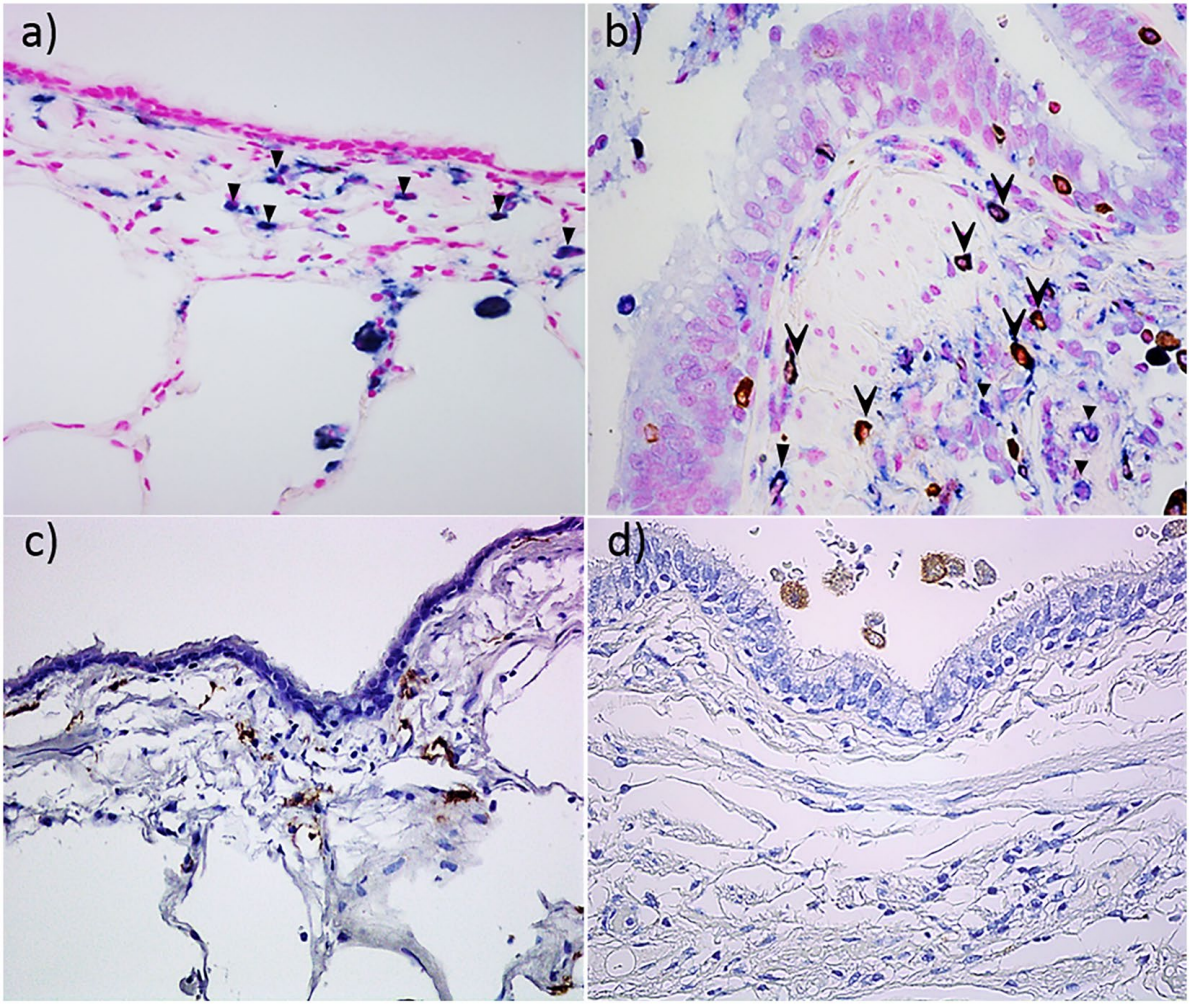
Representative micrographs of M1 macrophages dual stained for iNOS (brown) and CD68 (blue). (**a**) Thin epithelium and thin walled normal control, (**b**) thick epithelium and thick walled COPD-CS, counterstained with nuclear fast red (pink). () Dual stained CD68 + iNOS + cells, () only CD68 + cells. CD163 staining M2 macrophages (**c**) NC (**d**) COPD-CS.

A descriptive illustration comparing the macrophage sub-population phenotypes in NC and COPD-CS is represented in Fig. 2.

**Figure 3:**
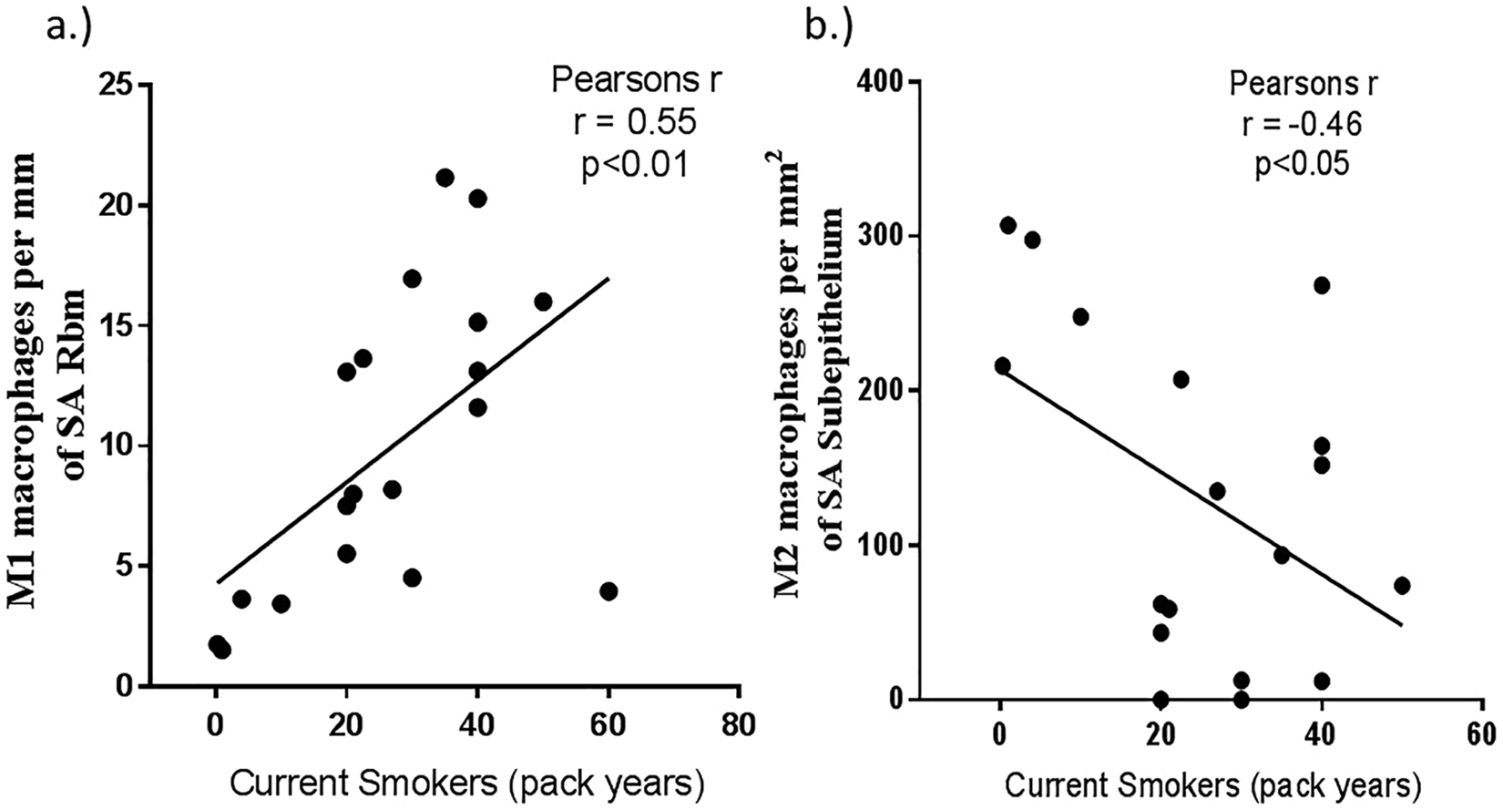
Regression analysis for tissue macrophage phenotypes with pack year history for NLFS and COPD-CS for (**a**) epithelial M1 and (**b**) sub-epithelial M2 macrophages.

Further, there was a positive correlation between smoking pack-years and increase in M1 macrophages in the epithelium (Pearsons r = 0.55, p = 0.006) (Fig. 3a.) while a negative correlation was observed for sub-epithelial M2 macrophages (Pearsons r = −0.46, p = 0.02) (Fig. 3b).

### Arginase-1 (Arg-1) expression in the SA wall

**Figure 4:**
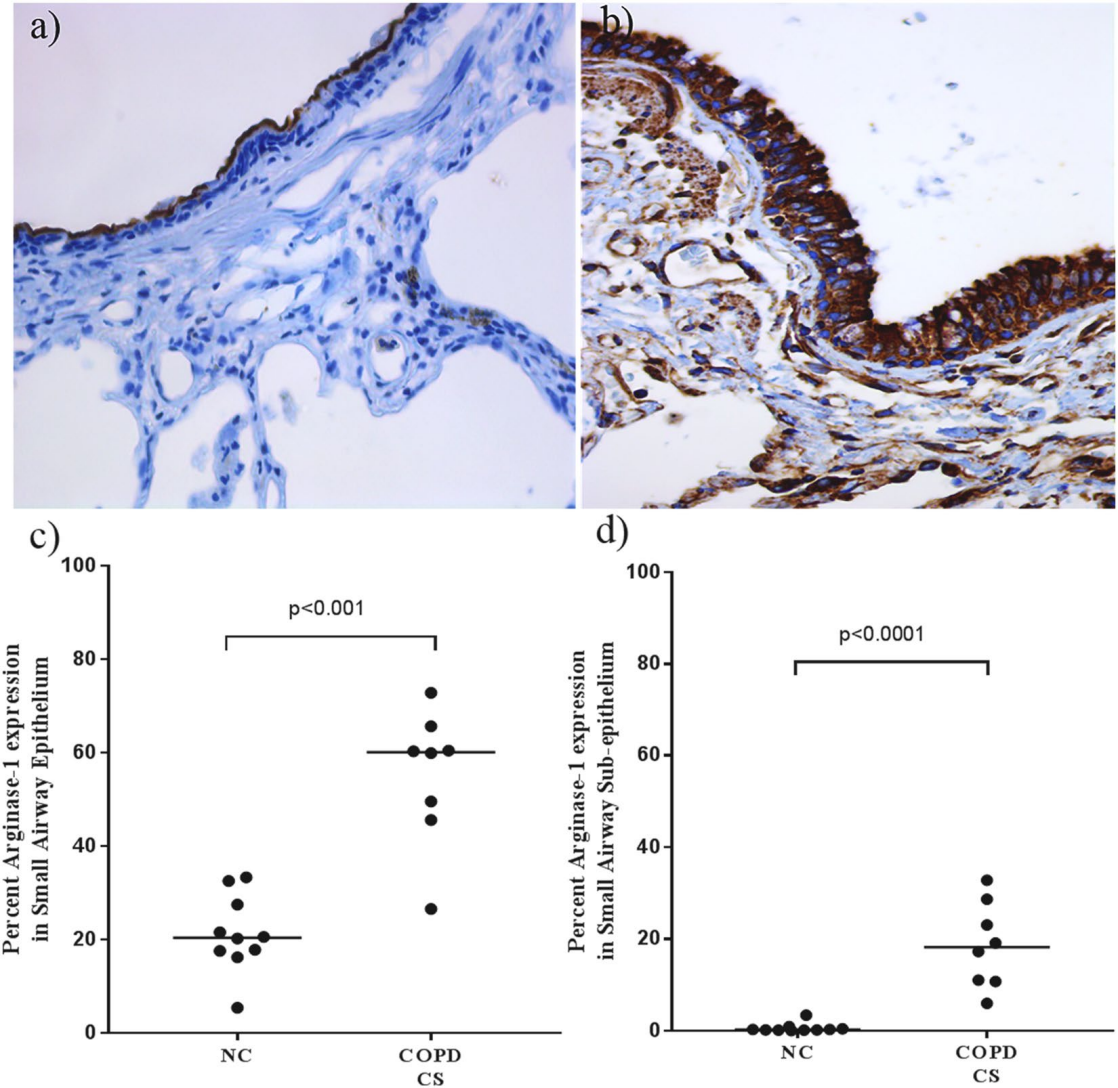
Arginase-1 expression in the small airway wall of (**a**) NC compared to (**b**) COPD-CS. A significant increase in Arginase-1 expression was observed in both (**c**) epithelium and (**d**) sub-epithelium. Data are presented as median and range; group comparisons with Mann-Whitney two-tailed t-test; p < 0.05 was considered significant.

The small airways wall tissue of COPD patient-CSs showed a marked overall non-specific increase in tissue expression of Arg-1, both in epithelium (p < 0.01) and subepithelium (p < 0.001) in comparison to normal controls (Fig. 4). At this stage we have not quantified this in non-COPD smokers, but descriptively the staining is present but less abundant.

### AM phenotypes in the Bronchoalveolar lavage (BAL)

**Figure 5:**
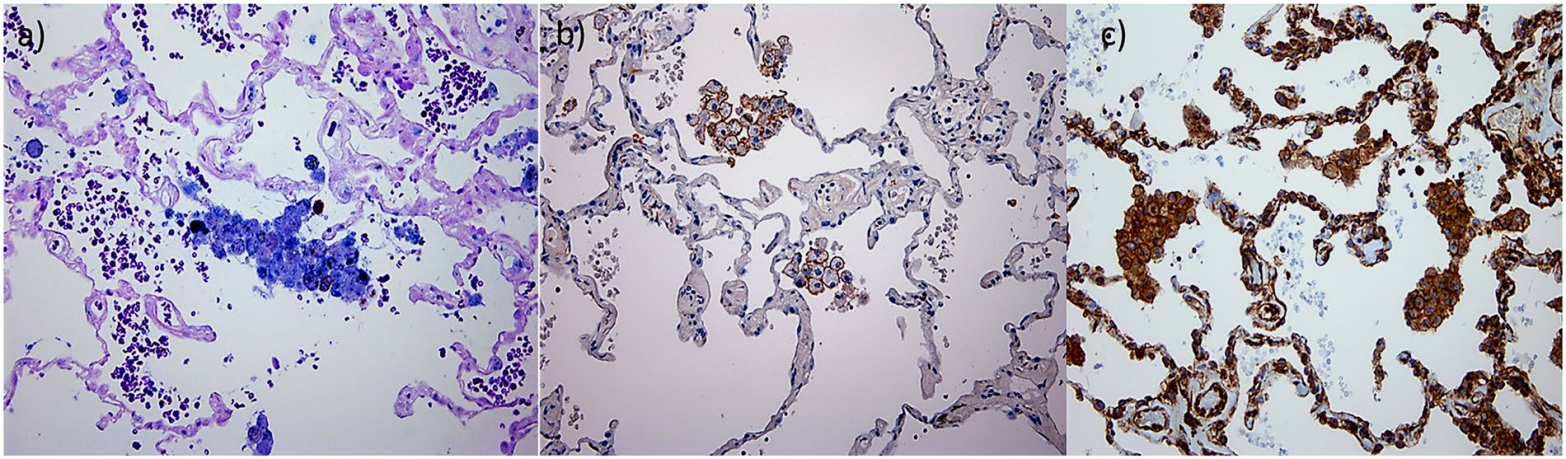
Expression patterns of alveolar macrophages (AMs) in the alveolar spaces in resected tissue of COPD patients (200x), (**a**) M1 AMs dual stained for iNOS (brown) and CD68 (blue), counterstained with nuclear fast red (pink), M2 phenotype macrophages stained brown with (**b**) CD163 and (**c**) arginase-1(ARG-1), counterstained with nuclear-stained hematoxylin (blue).

**Figure 6:**
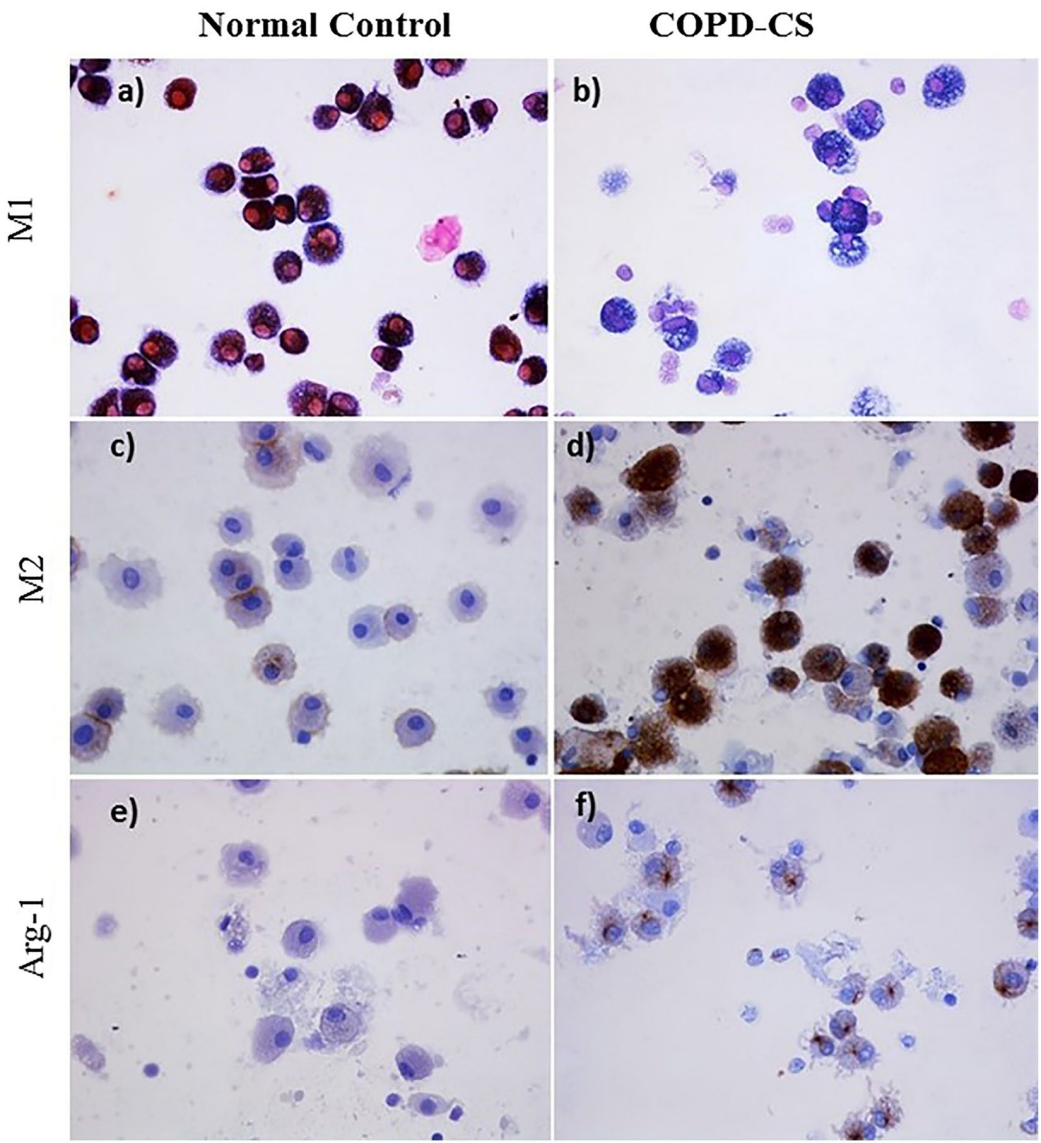
Representative pictures (400x) of M1 AMs dual stained for iNOS (brown) and CD68 (blue); (**a**) Normal control, and (**b**) COPD-CS, counterstained with nuclear fast red (pink). M2 phenotype macrophages stained brown with CD163 and arginase-1(ARG-1), (**c**,**e**) Normal controls and (**d**,**f**) COPD-CS respectively, with nuclear-stained hematoxylin (blue).

**Figure 7:**
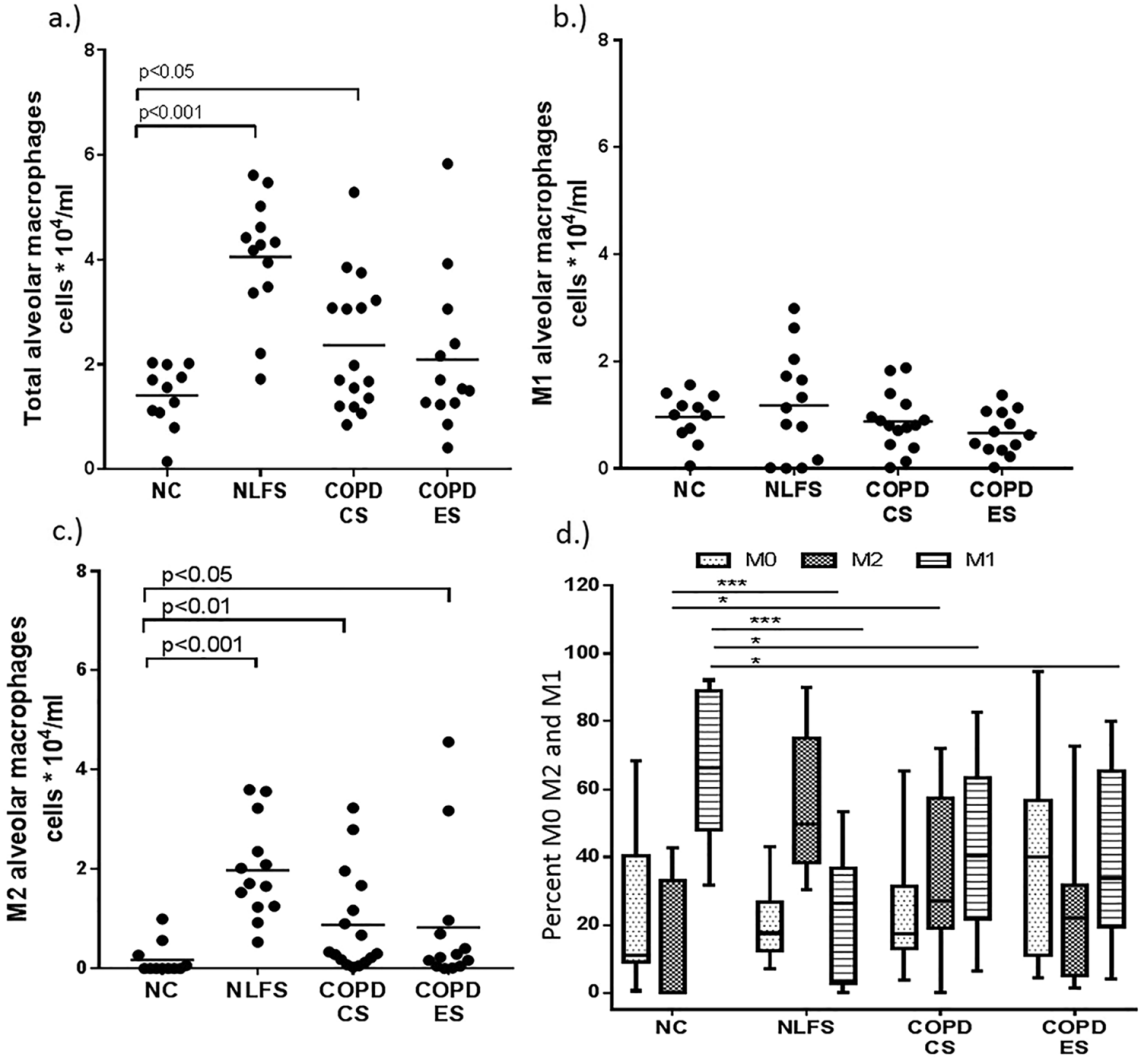
AMs numbers in BAL. (**a**) Total AMs, (**b**) M1 and (**c**) M2 macrophages in NC, NLFS COPD-CS, and ES. (**d**) Represent percent of total macrophage for each phenotypic population (Data in median and range; ***p < 0.001, *p < 0.05).

When comparing the alveolar macrophages within the alveolar spaces (Fig. 5a–c) of resected lung tissue (also containing small airways) of COPD patients, we observed similarity in both morphological and M1/ M2 expression patterns with luminal macrophages derived from BAL lumen (Fig. 6b,d and f). However, we have provided here only the quntitative results frrom macrophages from the BAL samples. A two to three-fold increase in total BAL CD68 + AMs was found in NLFS (p < 0.01) and COPD-CS (p < 0.05), while in COPD-ES they were similar to normal levels (Fig. 7a). Unlike the tissue macrophage data, there was fewer undifferentiated percent M0 AMs across the groups (Fig. 7d).

The BAL AMs in NC were predominantly M1 (median percentage 66.3%; range 31.5–91.2) with essentially fewer M2s (median percentage 0% range 0.0–42.7) (Fig. 7d). There was a marked change in phenotype profiles in the clinical groups, with a decrease in the percent of M1 (median percentage 26.3%; range 0.1–53.1) in NLFS, COPD-CS (median percentage 40.4%; range 6.2–82.5) and ES (median percentage 33.6%; range 4.0–80) and increases in M2 macrophages (median percentage 49.5%; range 31.5–91.2) in NLFS, COPD-CS (median percentage 27.15%; range 0.2–71.9) and COPD-ES (median percentage 21.9%; range 1.3–72.7) (Fig. 5d).

**Figure 8:**
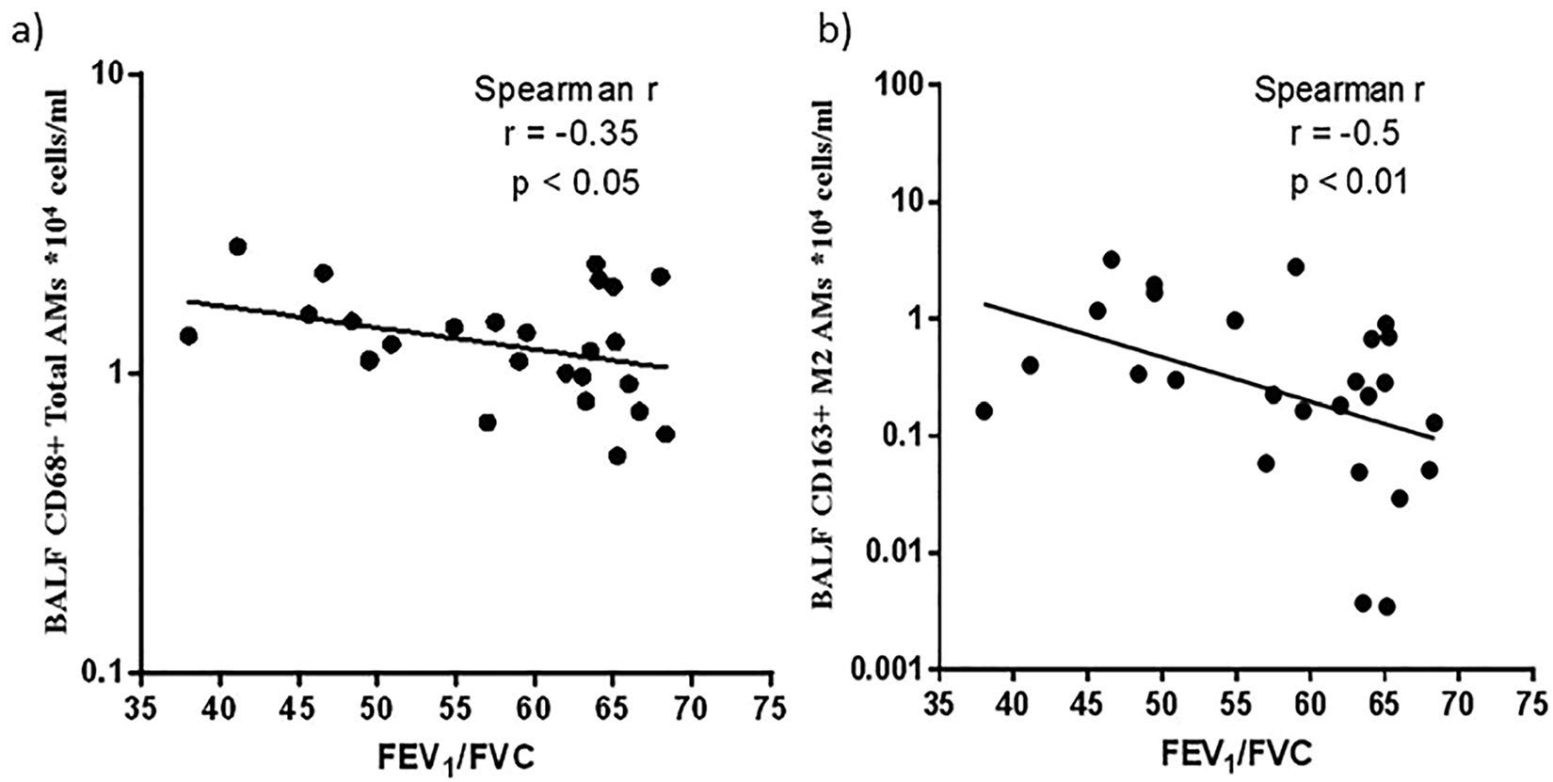
Regression analysis for BAL AMs with lung function in COPD groups. (**a**) Total AMs and (**b**) M2 AMs.

Both total CD68 + AMs (Spearman’s rho (rs) = −0.35, p =  < 0.05) and M2 AMs (Spearman’s rho (rs) = −0.5, p < 0.01) (Fig. 8a and b) correlated negatively with FEV1/FVC.

### BAL M2-associated cytokines were increased in COPD

**Figure 9:**
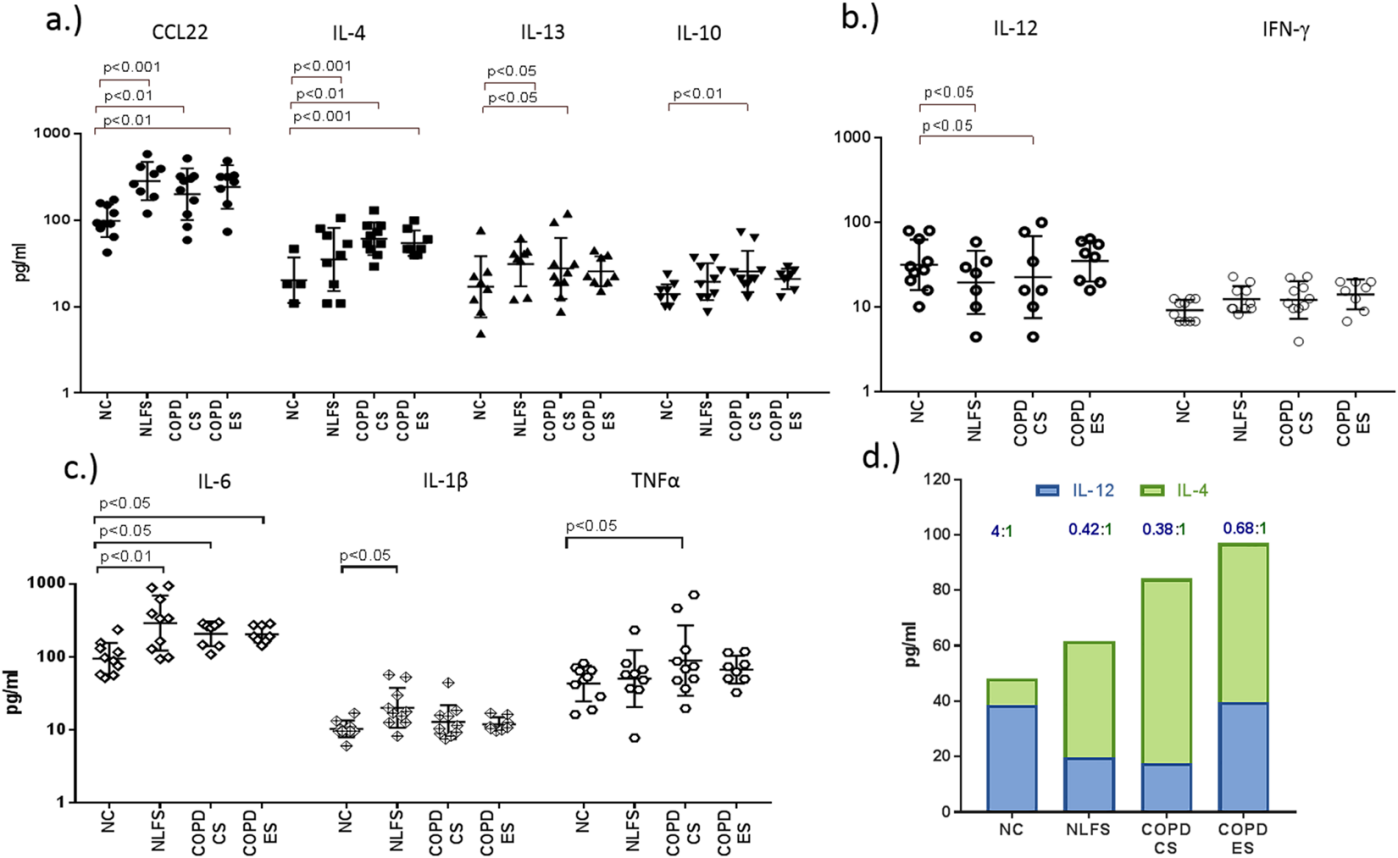
Cytokine profiles in BAL of NC, NLFS, COPD-CS and ES: (**a**) M1, (**b**) M2, (**c**) inflammatory cytokines (IL-6, IL-1β and TNFα) (**d**) IL-12/IL-4 ratio.

**Figure 10:**
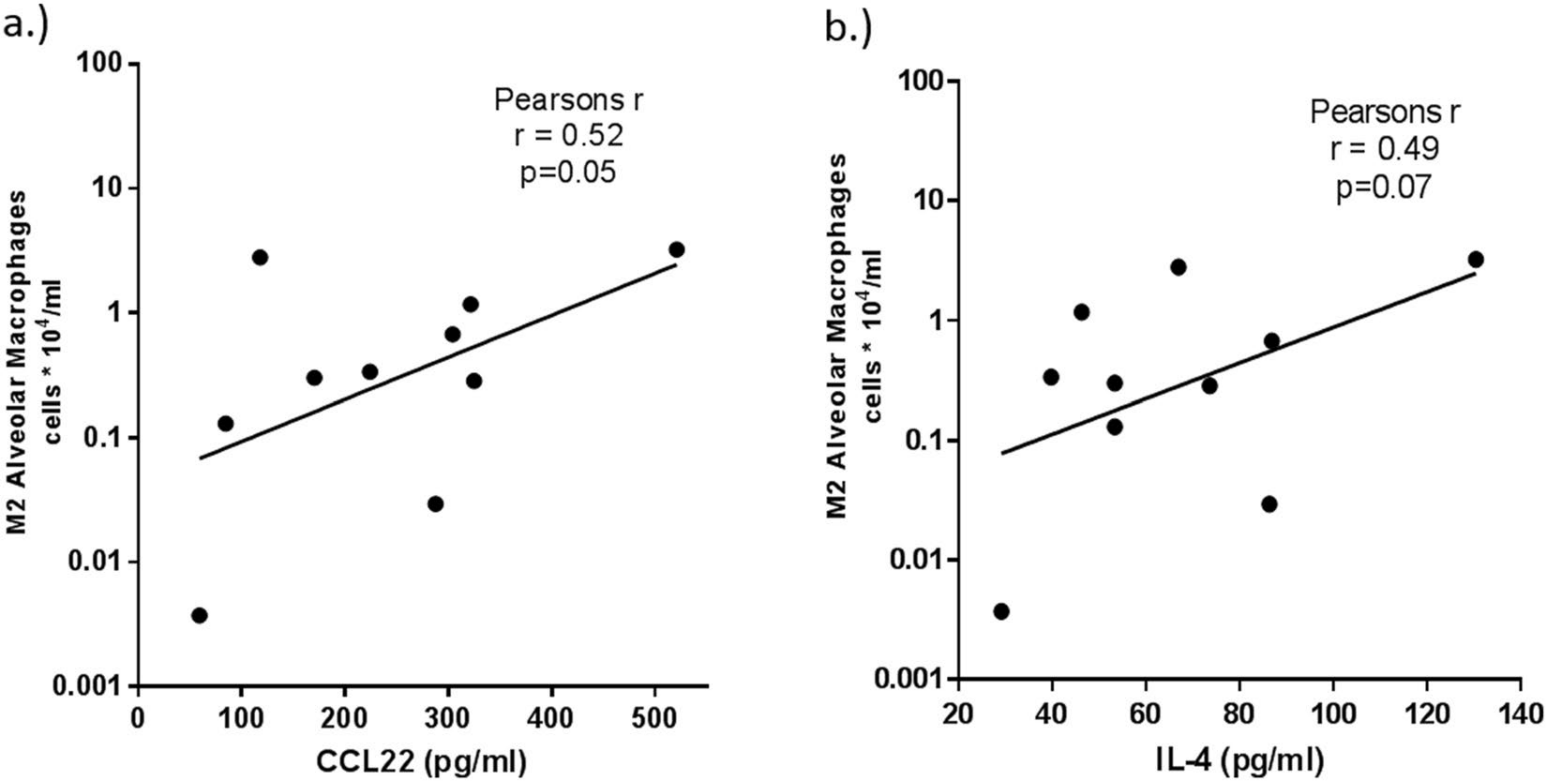
Positive correlation of M2 macrophages with (**a**) CCL22 and (**b**) IL-4 in COPD-CS.

A marked increase in M2-related MDC/CCL22, IL-4, IL-13, and IL-10 was observed in the BAL supernatants from NLFS and COPD subjects (Fig. 9a), with a decrease in the M1-related cytokine IL-12p40 but not for IFN-γ (Fig. 9b). We also found a significant increase in the pleiotropic cytokine IL-6 in both smoker and COPD groups (Fig. 9c). Further, a small but significant increase in proinflammatory IL-1β was observed in smokers but not in COPD while for TNFα there was an increase only in COPD-CS compared to normal controls (Fig. 9c). An increase in the ratio of IL-12p40 to IL-4 (M1/M2 cytokines) confirmed the switch to M2 dominance in BAL COPD-CS (Fig. 9d). There was a positive correlation in COPD-CS between CCL22 and IL-4 and M2 macrophage numbers (Fig. 10 a and b).

### Whole mouse COPD lung mRNA expression

**Figure 11:**
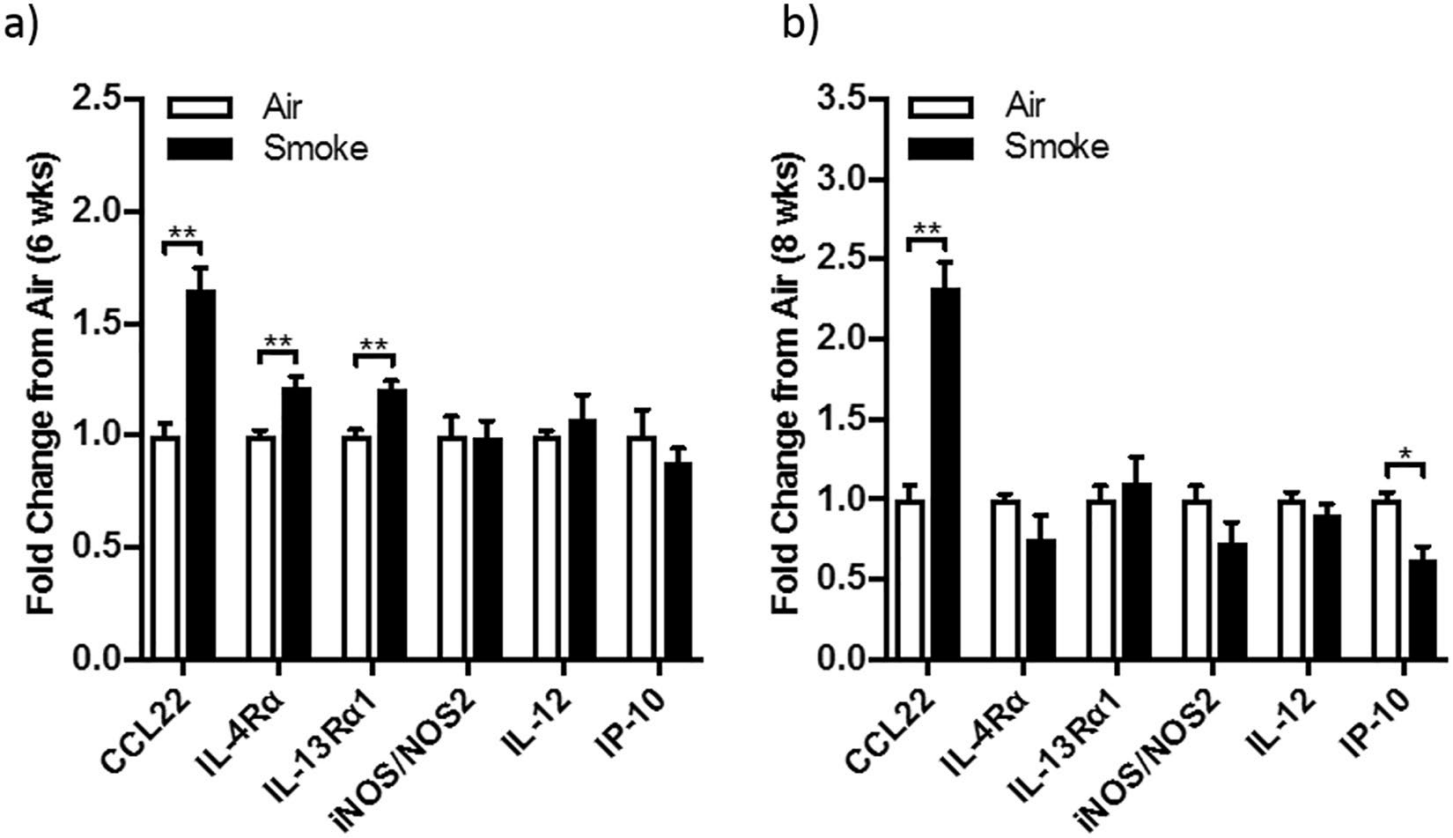
Fold changes in mRNA levels of M1 and M2 chemokines in the lungs of mice chronically exposed to cigarette smoke for (**a**) six and (**b**) eight weeks and normal air exposed controls (**p < 0.0.01; *p < 0.05).

We confirmed a similar pattern of M2 cytokine mRNA predominance in a well-established mouse model of chronic cigarette smoke exposure that is representative of exposure in a pack-a-day smoker^13,18–24^. We found with upregulation for CCL22 (fold change [FC] = 1.718; p = 0.012), IL-4Rα (FC = 1.270; p = 0.018), and IL-13Rα1 (FC = 1.673; p = 0.018), while there were no changes for M1-related iNOS/NOS2, IL-12 or IP-10 mRNAs (Fig. 11).

## Discussion

This study is the first to phenotypically differentiate small airway wall and airway lumen macrophage subpopulations based on their M1 and M2 phenotypes in normal, smokers and COPD patients. Our observations suggest dynamic differential switching in both the small airways and BAL AMs, but qualitatively these were quite different. In small airways, there was a switch towards a predominantly M1 phenotype in both NLFS and COPD-CS compared to to NC (Fig. 2) that suggests a smoking effect, while, in BAL AMs there was a switch towards M2 dominance (Fig. 6). Further, cytokines in BALF from smokers and COPD-CS were skewed towards a M2 profile. Data obtained from a chronic cigarette smoke-induced murine model also suggested that an M2 milieu in the present in whole lung tissue.

M1 and M2 macrophages are considered to be functionally differentiated, with M1 more pro-inflammatory and M2 more pro-fibrotic. For the M1 phenotyping, we chose iNOS + as a marker^25^ with CD68 co-staining to differentiate them from other iNOS producing cells such as dendritic and natural killer (NK) cells. For M2 we used CD163, which is a scavenger receptor that is upregulated in a more TH2 microenvironments^10,26^. Whether the different phenotypic skewing is a compartmental effect or caused by the movement of differentiated cells from wall to lumen needs further investigation. We believe that it is most likely the former as there was no gradient towards the lumen from sub-epithelium to epithelium, and there was no reciprocal decrease in wall cell numbers contributing to the significant increase in luminal macrophages.

The normal predominance of iNOS-expressing M1 macrophages occurs to fight pathogens through luminal production of nitric oxide (NO), an innate immune effector. However, such a response is non-specific, and when uncontrolled can cause considerable damage to host tissues and cells. Our observation of reduced percent M1 macrophages in the airway wall in COPD current and ex-smokers compared to normal lung function smokers, therefore, suggests a reduced ability fight infection^27^. It may also reflect homeostatic adaptation to avoid excessive tissue damage. Recent evidence suggests that elevated levels of NO inherently suppress the M1 phenotype^28^.

Given the iNOS changes, we also wished to investigate the functionally reciprocal Arg-1. Interestingly, we found a higher non-specific expression of Arg-1 throughout the airway wall mucosa including epithelium, sub-epithelium and alveolar septae in COPD-CS compared to normal non-smoker controls. This overexpression of Arg-1 could be the consequence of increased cellular catabolic activity, associated with increased oxidative stress in smokers, catalyzing L-arginine to urea and L-ornithine via urea cycle. L-ornithine is a known precursor to L-proline, a key amino acid in the biosynthesis of collagen, and associated with wound healing^15^. The excess deposition of collagen, however, leads to airway wall stiffness of the small airways, an important pathophysiological feature in COPD^29^. Further, exhaled NO is also well known to be decreased in smokers, but the reason for this has been unclear^30,31^.

Our finding of an M2 predominance in the airway luminal macrophages in smokers with and without COPD are resonant of two previous studies, although they found an increase percentage of M2 AMs only in COPD-ex smokers^10,32^. One study^32^ lacked normal control BAL, and neither assessed the M1 phenotype populations. Our findings are in line with the study by Shaykhiev *et al*.^33^, where gene expression analysis of cytokines and chemokines revealed more M2-polarised AMs in smokers and COPD compared to non-smoking controls. Further, our increase in both total AMs and especially M2 macrophage subtypes correlated to airflow obstruction, suggesting biological plausibility.

Our BAL cytokine data reflect the cellular phenotype switching that we observed in that compartment. IL-4 and IL-13 share a common receptor, IL-4Rα/IL-13Rα1, which signals via the JAK-1/STAT6 pathway, to induce differentially-activated M2 macrophages^34^. Studies by Rutschman *et al*.^35^, showed that the induction of the STAT6 pathway by IL-4 and IL-13 suppressed iNOS expression, and so NO production, by post-transcriptional modification. Other studies have also demonstrated that IL-4, IL-13, and IL-10 also synergistically upregulate Arg-1 expression in macrophages^36^. Our current study indirectly corroborates all these findings, but now in humans with the clinical disease.

We also observed an increase in CCL22 in BAL in NLFS and COPD. CCL22 is a regulatory chemokine secreted by M2 macrophages in response to TH2 polarized cytokines such as IL-4, IL-5, and IL-13, while it is downregulated by the TH-1 cytokine IFNγ^37,38^. Importantly, given the common co-association of COPD and lung cancer, CCL22 has been implicated in tumorigenesis. Its active secretion by M2 tumor-associated macrophages (TAMs) is known to promote malignancy by inhibiting suppressor T cell recruitment. Similar tumorigenic effects have been attributed to IL-6, which we also found to be elevated in smokers and COPD. Further, *in vitro* studies in macrophages also suggested that IL-6 promotes an M2 phenotype and increased M2-associated markers such as Arg-1^39^.

The differences in the polarization of macrophage subtypes between small airways and lumen were marked. This suggests a difference in the cytokine milieu in each anatomic microenvironment, promoting a shift towards the M1 phenotype in the airway wall but towards M2 in the airway lumen. This may have important implications for the distinct pathologies observed in each site in COPD disease, i.e. infection and ROS-induced innate immune activation in the lumen, but fibrosis and thickening of the airway wall. However, there are limitations to the current study, with tissue originating from two separate patient groups with BAL from COPD-CS, COPD-ex-smokers, NLFS and NC and small airway resections from similar physiological cohorts but cancer patients. Thus, it is possible that the macrophage phenotypes and cytokine profile is possibly influenced by the presence of cancer but we have quantified the airway wall tissue macrophages well away from cancerous areas.

We further investigated whether our human findings could be confirmed in a mouse smoking model and evaluated the mRNA levels in whole lung tissue of mice with cigarette smoke-induced experimental COPD. Interestingly, after six-weeks of smoke exposure, which represents an early stage of disease pathogenesis, mouse lung data confirmed the human findings, with specific M2 phenotypic mRNA for CCL-22, IL-4Rα, and IL-13α, with no changes in mRNA for M1 markers iNOS, IL-12p40 and IP-10. Our findings are currently limited to the mRNA from whole lung tissue and thus, unlike the human data, are not compartmentalized. However, we believe that the addition of mouse data in the current study strengthens our human findings and will be an impetus to our future mechanistic and interventional mouse studies about macrophage function.

## Conclusion

The major novel findings in this study are the reduction in M2 and increase in M1 macrophages in the airway wall of SAs in smokers and COPD. M1 are the strongest signal for innate inflammatory up-regulation we have seen in the airway wall to date. The finding of an M2 switch in BAL in smokers with and without COPD was in stark contrast to the macrophage phenotype in the small airway wall. BALF cytokine profiling revealed promotion of an M2 phenotype. The switching was confirmed in lung tissue from a chronic smoking mouse model. The overall tissue expression of Arg-1 in the SA wall suggests increased catabolic activity, a sign of cellular senescence but could also have implication for in lung fibrosis and airway resistance. These novel findings are potentially important in understanding the pathophysiology of the respiratory tract’s response to smoking and in the etiology of COPD; they need to be taken into account when considering mostly unexplained cellular functional phenomena associated with COPD, and the specific vulnerability of COPD sufferer to lung cancer.

## Methods

### Ethics approval

The Tasmanian Health & Medical Human Research Ethics Committee approved the study (EC00337- Small Airway resected tissue and H6532 – BAL fluid samples). Informed consent were directly obtained from all participating subjects. The Animal Ethics Committee of The University of Newcastle, Australia approved all mouse related protocols. All experiments included in this study are in accordance to the relevant institutional guidelines and regulations.

### Subject classification

#### SA resected tissues

Forty patients consented for inclusion in this study Table 1. Subjects all had primary non-small cell lung cancer, with an approximately equal distribution of squamous and adenocarcinoma, and consented for their surgical tissue to be used for research at Royal Hobart Hospital. Twenty patients had demonstrated mild-moderate, Global Initiative for Obstructive Lung Disease (GOLD) stage I and II COPD of which nine were COPD-CS and eleven COPD-ES (>1year smoking cessation). Eleven individuals NLFS. Ten non-smoking tissues were obtained from the James Hogg Lung Registry, the University of British Columbia with approval from the Providence Health Care Research Ethics Board H00–50110, and were included as a control group (NC) for comparison. Subjects with other respiratory diseases, a history of recent acute exacerbation of COPD and those on systemic or inhaled corticosteroids were excluded from the study. The surgically resected material was taken well away from the primary tumor and contained non-cancer affected small airways.

**Table 1:** Demographic details and lung function data for participants.

Groups	NC		NLFS		COPD-CS		COPD-ES	
Tissue source	BAL	SA	BAL	SA	BAL	SA	BAL	SA
Subjects (n)	11	10	13	11	16	9	13	11
Age (years)	44 (20–68)	68 (63–75)	50 (30–66)	70 (52–79)	61 (46–78)	64 (59–78)	62 (53–69)	68 (56–85)
Smoking (Pack-Years)	0	0	32 (10–57)	33 (3–60)	45 (18–78)	28.5 (2–50)	51 (18–150)	33 (18–36)
FEV_1_ /FVC (post BD)^†^	82 (71–88)	N/A	77 (70–96)	76 (70–90)	59 (46–68)	66 (60–70)	57 (38–68)	64 (55–69)

Data represented here as median and range in brackets.

NC- Normal non-smoker controls, NLFS- Normal Lung Function Smokers, COPD-CS- COPD-Current Smokers and COPD-ES- COPD-Ex Smoker.

BAL- Bronchoalveolar Lavage; SA- Small Airways from resected tissues.

FEV_1_/FVC- Ratio of the forced expiratory volume in the first one second to the forced vital capacity of the lungs.

^†^Post BD values after 400 µg of salbutamol.

N/A**-** Not Available.

#### BAL Fluid (BALF)

Fifty-four human subjects volunteered for the study. BALF from 13 NLFS, 16 COPD-CS, and 14 COPD-ES were compared with 11 NCs. Subjects with recent respiratory disease, infection or acute exacerbation of COPD and those on systemic or inhaled corticosteroids were excluded from the study. BALF was obtained and processed as described previously (14, 15). Once extracted the BALF was transported to the laboratory at 4 °C for processing and analysis. Total cell counts were determined on the extracted unfiltered BALF using a hemocytometer, and further 200 µL of the BALF was cytocentrifuged at 100 g for 5 min to produce two cytospots on a glass slide. BAL cytospins were fixed in formalin for 10 minutes before staining. BALF supernatants were prepared by filtering the BALF through a 200-micron mesh and centrifuging at 250 g for 15 min at 4 °C to remove cell debris. Aliquots of the BALF supernatants were stored at −80 °C until used.

### Immunohistochemistry

Resected tissue was formalin fixed, paraffin embedded, tissue sections were cut at 3.5-micron thickness, and were dewaxed and rehydrated in ethanol. Further, both BAL cytospins and the resected tissues underwent heat retrieval and endogenous peroxidase activity blocked with 3% hydrogen peroxide for 10 minutes.

For M1 macrophages, resected tissues and BALF cytospins were dual stained with mouse anti-CD68 monoclonal antibody (KP1, Dako, M0814, 1/400 dilution) and a rabbit anti-iNOS polyclonal antibody (Thermo Fisher Australia, PA1–21054, 1/100 dilution). Bound iNOS antibodies were elaborated using peroxidase-labelled Rabbit Envision + and visualized as brown using 3-3′-Diaminobenzidine (DAB) (K3468; Dako Denmark A/S), while CD68 antibodies were developed using Dako REAL detection system (K5005; Dako) and visualized as blue with BCIP/NBT (5-bromo-4-chloro-3′-indolyphosphate and nitro-blue tetrazolium) (K0598; Dako) in a ready-made substrate system. Endogenous alkaline phosphatase activity was inhibited by the addition of Levamisole (X3021; Dako). Further, the slides were counterstained with nuclear fast red to visualize pink nuclei. Single stained CD68 + cells negative for iNOS were considered as the M0 cell population.

To characterize M2 macrophages, CD163s and Arginase-1 staining were performed using mouse anti-CD163 (EDHu-1, AbD Serotec, MCA1853, 1/100 dilution) anti-Arg1 (BD Biosciences, 610708, 1/100 dilution) antibodies, for 90 minutes. Bound CD163 and Arg1 antibodies were elaborated using peroxidase-labelled mouse Envision + (Dako Denmark), developed with DAB and were counterstained with hematoxylin.

### Image Analysis

Computer-assisted image analysis was performed with a Leica DM 2500 microscope and Leica DFC495 camera and Image Pro Plus 7.0 software. Five random fields selection of small airways less than two mm in thick (a minimum of two airways per subject) were chosen for comprehensive analysis without *ad hoc* area selection, although muscle bundles and glands were excluded from the area surveyed. Small airways sub-epithelium up to 100 microns deep were quantitated. Stained M0, M1 and M2 cells in the sub-epithelium and epithelium were separately counted and is presented here as per mm^2^ of the area surveyed and per mm of reticular basement membrane (RBM) length respectively. For Arg-1 expression, separate analysis was done for the total sub-epithelium (excluding muscle areas) and epithelium and further, the data here is represented as percent of tissue Arg-1 expression.

BAL cells counts were done using brightfield microscope (Olympus BX53) assisted by Visiopharm newCAST™ software. An automated motorized system provided an unbiased uniform random area sampling for 12 fields per cytospot, and stained cells were manually counted for each selected field. Counts were normalized with BAL dilution factor and presented here as cells per ml of the original BAL sample.

### Cytokine Analysis

Cell-free BALF supernatants were thawed on ice and concentrated ten fold, using 3 kDa cutoff Amicon® Ultra-4 Centrifugal Filter Units (UFC800308 Merck Millipore) and centrifugation (2000g, 30 min, 4 °C). Human cytokines for M1/proinflammatory cytokines (IL-12, IFNγ, IL-1β and TNFα) and M2 (IL-4, IL-13, CCL22, IL-10, IL-6) macrophages were quantitated using multiplexing (MPHCYTOMAG60; Millipore Multiplex kits), and analysis was done using Luminex™ MagPix Multiplex technology platform according to manufacturer instructions. Chemokine/cytokine quantitation was derived from the standard curve and represented here as picograms per ml of original BAL sample.

### mRNA analysis of mouse lung

Mice were exposed through the nose-only to the smoke of 12 research grade cigarettes, for one hour, twice per day, five days per week, for six weeks^20,21,28^. This equates to a pack-a-day human smoker. When early features of COPD developed after six weeks, lung mRNA was extracted, and microarrays performed using Affymetrix GeneChips. GeneSpring analysis was employed to assess the mRNA transcript levels of M1 (IP10, IFN-γ, IL-12) and M2 markers (CCL22, IL-4Rα, IL-13Rα).

### Statistical analysis

Following a check for normal distribution, the analysis here is represented as median and range, non-parametric (Kruskal–Wallis) analysis of variance with multiple comparisons using Dunn’s test. Linear regression and Pearson or Spearman r’ was used for correlation analysis. Mouse data were analyzed using unpaired t-tests assuming Gaussian distribution with PRISM V6.0d software (GraphPad, La Jolla, CA, USA), and are presented as means ± SEM of 4–8 mice/group, p < 0.05 was considered statistically significant.
